# How a Subclinical Unilateral Vestibular Signal Improves Binocular Vision

**DOI:** 10.3390/jcm12185847

**Published:** 2023-09-08

**Authors:** Frédéric Xavier, Emmanuelle Chouin, Véronique Serin-Brackman, Alexandra Séverac Cauquil

**Affiliations:** 1 Sensory and Cognitive Neuroscience Unit LNC UMR 7231 CNRS, Aix-Marseille University, St-Charles, 3, Place Victor Hugo, 13003 Marseille, France; 2 Pathophysiology and Therapy of Vestibular Disorders Unit GDR 2074, Aix-Marseille University, St-Charles, 3, Place Victor Hugo, 13003 Marseille, France; 3 Medical, Maieutics and Paramedical Department, Faculty of Health, University Toulouse III, Paul Sabatier, 31062 Toulouse, France; 4 ActiVest—Vestibular Functional Exploration in Humans and Non-Human Primates Unit GDR 2074, St-Charles, 3, Place Victor Hugo, 13003 Marseille, France; 5 Brain and Cognition Research Center CerCo UMR 5549 CNRS, University Toulouse III, Paul Sabatier, 31062 Toulouse, France

**Keywords:** galvanic vestibular stimulation, disconjugate eye movements, stereoscopic vision, vestibular error signal

## Abstract

The present study aimed to determine if an infra-liminal asymmetric vestibular signal could account for some of the visual complaints commonly encountered in chronic vestibular patients. We used infra-liminal galvanic vestibular stimulation (GVS) to investigate its potential effects on visuo-oculomotor behavior. A total of 78 healthy volunteers, 34 aged from 20 to 25 years old and 44 aged from 40 to 60 years old, were included in a crossover study to assess the impact of infra-liminal stimulation on convergence, divergence, proximal convergence point, and stereopsis. Under GVS stimulation, a repeated measures ANOVA showed a significant variation in near convergence (*p* < 0.001), far convergence (*p* < 0.001), and far divergence (*p* = 0.052). We also observed an unexpected effect of instantaneous blocking of the retest effect on the far divergence measurement. Further investigations are necessary to establish causal relationships, but GVS could be considered a behavioral modulator in non-pharmacological vestibular therapies.

## 1. Introduction

In the United States, 10 million patients seek medical consultations for vertigo each year [1]. According to various authors, this number could extend to 20 million individuals, including 3.9 million cases requiring emergency hospital visits [2], accounting for approximately 3.3% to 4% of total visits to these services (3.3% [3], 3.5% [2], 4% [4]). In 2019, Hulse published a one-year prevalence of vertigo in Germany of 6.5%. Among the 70,315,919 patients included in the study, 3,406,169 (4.8%) were categorized with non-specific dizziness, and 1,137,294 patients (1.6%) were categorized with peripheral vestibular disorders [5]. Patients’ complaints are highly heterogeneous and significantly impact their quality of life. One of the most common complaints is visual discomfort experienced during movements, such as the sensation of blurred vision, vertigo in situations of intense visual flow, like in the presence of crowds in department stores, and visual fatigue during reading or screen use.

Vision plays a crucial role in spatial orientation and balance by detecting environmental variations [6,7]. Working in synergy with the vestibular system (inner ear) and the somesthetic proprioceptive system (sensory receptors of muscles and joints), it contributes to maintaining body stability and coordination [8]. Visual processing starts with photoreception in the retina and is achieved at different levels of the cerebral cortex, allowing the central nervous system to distinguish shapes, colors, movements, and distances, so as to elaborate mental representations of our environment [9].

The vestibular system differs from other sensory systems in three distinct aspects: i/the existence of “vestibular noise”, referring to random and unwanted fluctuations in sensory signals from the vestibular system [10]; ii/the permanent asymmetry of the bidirectional signal (or relative vestibular bias) weighted by somesthesis and vision [11]; and iii/the detection and discrimination thresholds corresponding to the extraction of a suprathreshold signal. The suprathreshold signal must be understood as the extraction of a “clear” signal, either arising from the variability in a unilateral signal (e.g., during caloric stimulation) or from the summation of an ipsilateral excitability signal coinciding with a contralateral inhibitory signal, amidst the ongoing discharge of sensory cells or “vestibular noise” [12,13]. These concepts must be introduced because they allow for determining the physiological threshold beyond which a physical stimulus imposes an adaptive or behavioral response (i.e., avoidance strategies) [14]. In clinical practice, this threshold notion is well-established for exteroceptive senses such as hearing and vision [15]. For the vestibular system, determining thresholds is more complicated as the vestibular sense is generally implicit, operating automatically and unconsciously to maintain body balance and spatial orientation, and its output expression is multimodal. Detection thresholds are expressed by the absence or presence of motion perception, and discrimination thresholds distinguish discrepancies in velocities, angles, internal/external movements, etc. In the context of unilateral peripheral vestibular clinical cases, the suprathreshold signal can be likened to a vestibular error signal (VES), either due to reduced excitability (e.g., total neurotomy) or excessive excitability (e.g., VPPB). In otoneurological practice, the analysis of VES is limited to its subcortical modulation expression, clinically observable as visuo-perceptivo-motor manifestations [11,16]. However, studying the impact of a subthreshold VES could lead to new understandings of the complaints found in chronic patients. A subliminal VES can be present in slow-progressing pathologies such as vestibular schwannoma. Studying artificial VES is a good non-invasive way to highlight several VES profiles. In particular, studies using investigative vestibular implant (VI) approaches and galvanic vestibular stimulation (GVS) suggest that the vestibular system has robust adaptability to electric stimulations induced by this procedure [10,11,12,13]. This adaptability depends on the type and pattern of stimulation used, such as frequency modulation, amplitude modulation, cross-channel stimulation of one or multiple channels, etc. However, some stimulations may be deleterious [14,15,16,17,18,19,20] and lead to the reproduction of a suprathreshold VES. The clinical adaptive response is observed by the emergence of a static and dynamic symptomatology that is almost identical to what is observed in the case of a unilateral lesion. These studies also demonstrate that prolonged stimulation induced with VIs alters the way vestibular signals are integrated into the brain, similar to what occurs in neighboring structures during chronic unilateral vestibular lesions. This engagement of neural plasticity and disturbances in vestibular compensation suggests that a suprathreshold unilateral peripheral VES may have significant implications for the central integration of sensory information, disrupting the construction of internal models for perceiving the environment.

GVS consists in transcranial stimulation that can modulate vestibular afferences by inhibiting (anodic current) or stimulating (cathodic current) them [21,22]. By polarizing the peripheral loop (semicircular canals, otolithic organs, vestibular nerves, and vestibular nuclei), it affects balance, oculomotor function, and spatial orientation. The GVS effect is comparable to the clinically observable suprathreshold unilateral peripheral VES [23]. Many studies in the field show that GVS facilitates partial or complete neural connections, allowing for progressive recovery of lost vestibular function through synaptic circuit reorganization [24,25]. It also has a reweighting effect on the connection between vestibular pathways and the limbic system. Some authors found that GVS acts on all pathways involved in the vestibular system response [19,26,27,28]. Depending on the use of subliminal or supraliminal thresholds and the duration of stimulation, a VES effect was described, leading to modifications in the plasticity of vestibular and postural reflexes [19,26,27,28,29].

The present work investigates what happens to oculomotor indicators when a subthreshold VES (below discrimination thresholds) that does not generate measurable clinical manifestations is applied. This question is worth addressing since otoneurological consultations often encounter complaints that only partially correspond to the already established clinical model of unilateral peripheral deficit. We can draw parallels with unilateral hydrops or vestibular schwannoma, which induce an erroneous signal with slow and subthreshold progression due to 1/the high plasticity of peripheral vestibular synaptic circuits and 2/central modulation of detection and discrimination thresholds. The question of the effect of subthreshold GVS stimulation is relevant: can it modify any visuo-oculomotor indicators without perceptual and behavioral manifestations? Our study was undertaken to describe the visuo-oculomotor consequences of a subthreshold VES, artificially and transiently administered unilaterally with GVS in healthy subjects, to identify specific marker evolutions over time and assess the effect of aging on these phenomena.

## 2. Materials and Methods

### 2.1. Study Design

**Figure 1:**
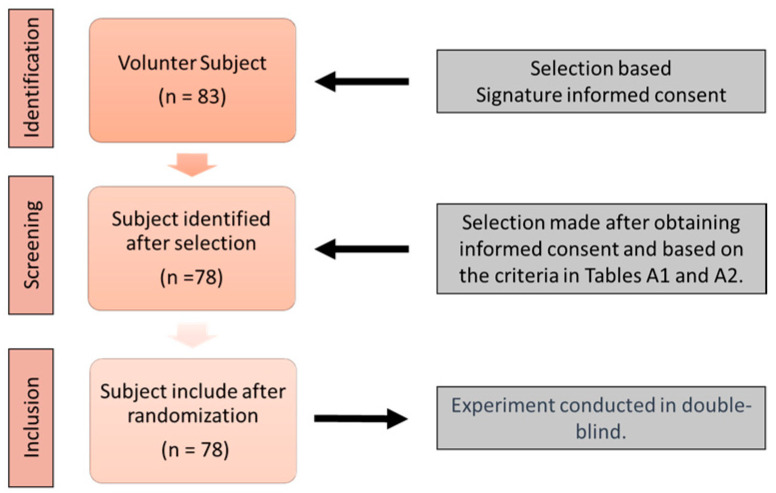
Flow diagram for subject inclusion in the stimulation test.

A crossover experiment was conducted at the Center for Brain and Cognition Research (CerCo) in collaboration with the Orthoptics School of Toulouse, France, from 2018 to 2022. Healthy male and female subjects aged between 18 and 60 years were recruited on a voluntary basis. The study was approved by the INSERM Ethics Evaluation Committee (INSERM n°14-155ter). Before participation, subjects read an information sheet and provided written consent. Subjects completed an initial questionnaire and orthoptic evaluation to verify their eligibility based on exclusion criteria (see Appendix A, Table A1 and Table A2). The inclusion procedure is described in Figure 1.

### 2.2. Experimental Protocol

Galvanic vestibular stimulation (GVS) was performed using a DIGITIMER DS-5 stimulator delivering a square wave signal with a maximum intensity of 1 mA through disposable adhesive electrodes. We chose a 1 mA intensity, for which we did not observe any consistent behavioral response in our experimental conditions. The stimulation protocol consisted of 10 bursts of 2 s, separated by 10 s, for a total duration of 120 s. Two categories of stimulations were performed (1) unilateral vestibular anodal stimulation on the right side (GVS) via one mastoid electrode and one cervical electrode (spinous process of C7) and (2) sham or control stimulation via two electrodes placed on both sides of the spinous process of C7. Eight orthoptics student operators conducted the manipulations, supervised by a senior to improve reliability, validity, control of variability, and reproducibility of measurements. Subjects were placed in a Romberg position on a flat surface. Optometry measurements were taken before (T0), during (T1), after (T2), and 15 min after the stimulation (T3). The measured follow-up indicators included: far convergence at 5 m (C), near convergence at 40 cm (C′), far divergence at 5 m (D), near divergence at 40 cm (D′), near point of convergence (PPC), far stereoscopic acuity at 2.5 m (Kratsa–Barron–Laraudogoitia), and near stereoscopic acuity at 40 cm (TNO; see Appendix A, Table A3). The subjects went testing twice, on 2 different days, and the order of GVS and sham stimulations was randomized to avoid biases.

### 2.3. Statistical Analysis

A baseline correction (T-T0) was applied to rule out the initial effect. Statistical analysis was performed using JASP software version 0.17.1. For each group of variables, a Shapiro–Wilk test was conducted to determine if the data approximately followed a normal distribution. A repeated measures ANOVA was used to determine whether the type of stimulation (GVS and/or sham) influenced the evolution of follow-up indicators over time based on the subjects’ age category. A sphericity test was conducted, and a Huynh–Feldt correction was applied when ε ≥ 0.75. A post hoc analysis with Student’s *t*-test was used with a Holm correction to adjust the significance level. The significance level for tests was set at *p* ≤ 0.05, and the Holm procedure was applied to adjust the significance level based on the number of independent comparisons (*p* < 0.012).

## 3. Results

### 3.1. Data Description

Thirty-four subjects aged 20–25 years and forty-four subjects aged 40–60 years were included. For each studied group, the distribution of variables according to a normal distribution was verified. The list of variables with a *p*-value between 0.05 and 0.1, as calculated using the Shapiro–Wilk test, is as follows:

D′5′ 20–25 years: mean 8.529; std. deviation 4.129; Shapiro–Wilk 0.954; *p* = 0.1.

C5′ 20–25 years: mean 17.941; std. deviation 6.532; Shapiro–Wilk 0.949; *p* = 0.1.

PPC15′ 40–60 years: mean 7.000; std. deviation 2.246; Shapiro–Wilk 0.970; *p* = 0.096.

Sham D′ 20–25 years: mean 9.294; std. deviation 3.186; Shapiro–Wilk 0.941; *p* = 0.064.

Sham D′ 40–60 years: mean 9.545; std. deviation 3.950; Shapiro–Wilk 0.963; *p* = 0.062.

Sham NPC 40–60 years: mean 6.795; std deviation 2.326; Shapiro–Wilk 0.964; *p* = 0.080.

Sham D′5′ 40–60 years: mean 9.273; std deviation 4.358; Shapiro–Wilk 0.953; *p* = 0.072.

Sham D′15′ 20–25 years: mean 8.529; std deviation 3.628; Shapiro–Wilk 0.949; *p* = 0.1.

The rest of the normality analysis yields *p*-values > 0.05. With the remaining data conforming to normality, the comprehensive analysis is available upon request.

### 3.2. Indicators Evolution According to the Stimulation Factor

#### 3.2.1. Near Convergence Indicator (C′; Figure 2A; Table 1)

The repeated measures ANOVA confirms that the variation in C′ measurements under GVS stimulation is statistically significant (F (2.613, 198.569) = 10.073; *p* < 0.001). The post hoc analysis reveals a significant mean difference using Student’s *t*-test between T0 and T2 (μ(T0)–μ(T2) = −2.407; *p* < 0.002); T0 and T3 (μ(T0)–μ(T3) = −3.432; *p* < 0.001); and T1 and T3 (μ(T1)–μ(T3) = −2.527; *p* < 0.001). Under sham stimulation, non-significant variation in C′ measurements is found (F (2.755, 209.389) = 2.358; *p* = 0.078). However, the post hoc analysis does not reveal significant links in the Student’s *t*-test (*p* > 0.007).

#### 3.2.2. Far Convergence Indicator (C; Figure 2B; Table 1)

Under GVS stimulation, the repeated measures ANOVA shows a significant variation in C measurements (F (2.772, 210.642) = 13.027; *p* < 0.001). The post hoc analysis reveals a significant mean difference in using Student’s *t*-test between T0 and T2 (μ(T0)–μ(T2) = −2.116; *p* < 0.001); T0 and T3 (μ(T0)–μ(T3) = −2.685; *p* < 0.001); T1 and T2 (μ(T1)–μ(T2) = −1.522; *p* = 0.007); and T1 and T3 (μ(T1)–μ(T3) =−2.092; *p* < 0.001). Under sham stimulation, the repeated measures ANOVA shows a non-significant variation in C measurements (F (2.492, 189.425) = 1.556; *p* = 0.208). The post hoc analysis does not reveal significant links in the Student’s *t*-test (*p* > 0.007).

#### 3.2.3. Near Divergence Indicator (D′)

**Figure 2:**
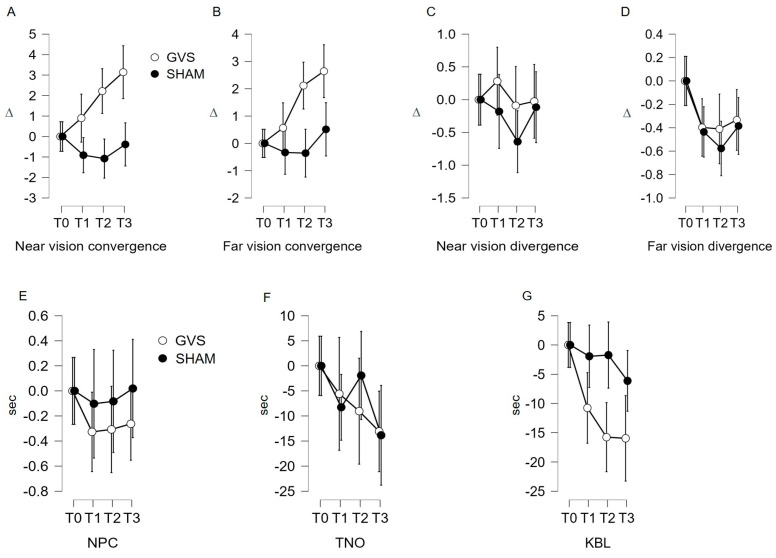
(**A**–**G**) Evolution of monitoring indicators according to the stimulation site. GVS: vestibular galvanic stimulation; C7: stimulation on either side of the C7 spine; NPC: near point of convergence; TNO: stereopsis with graded circle. Distance stereoscopy test (0.40 m); KBL: Kratsa–Barron–Laraudogoitia. Distance stereoscopy test (2.5 m); ∆: diopter, sec: second. The error bars indicate 95% confidence intervals.

The statistical analysis does not show a significant link (Figure 2C; Table 1).

#### 3.2.4. Far Divergence Indicator (D, Figure 2D; Table 1)

The variation measured in D under GVS stimulation fails to reach statistical significance (repeated measures ANOVA F (2.134, 162.208) = 2.942; *p* = 0.052) for the main effect and the post hoc analysis. In contrast, under sham stimulation, the repeated measures ANOVA shows a significant variation in D measurements (F (2.699, 205.090) = 7.641; *p* = 0.001). The post hoc analysis shows a significant link in the Student’s *t*-test between T0 and T1 (μ(T0)–μ(T1) = 0.460; *p* = 0.004) and T0 and T2 (μ(T0)–μ(T2) = 0.622; *p* < 0.001). The interval analysis T0–T3 (μ(T0)–μ(T3) = 0.013; *p* < 0.013) is debatable.

#### 3.2.5. Near Point of Convergence (NPC) and Stereopsis with Graded Circle (TNO) Indicators

The statistical analysis does not show a significant link (Figure 2E,F; Table 1).

#### 3.2.6. Kratsa–Barron–Laraudogoitia Indicator (KBL; Figure 2G; Table 1)

Under GVS stimulation, the repeated measures ANOVA shows a significant decrease in KBL measurements (F (2.959, 224.850) = 9.003; *p* < 0.001), which was also found in the post hoc analysis (Student’s *t*-test between T0 and T1 (μ(T0)–μ(T1) = 11.200; *p* = 0.012); T0 and T2 (μ(T0)–μ(T2) = 16.634; *p* < 0.001); and T0 and T3 (μ(T0)–μ(T3) = 16.955; *p* < 0.001). Under sham stimulation, the repeated measures ANOVA shows a non-significant variation in C measurements (F (2.526, 192.010) = 1.435; *p* = 0.238). The post hoc analysis does not reveal significant links in the Student’s *t*-test (*p* > 0.012).

### 3.3. Evolution of Follow-Up Indicators in Both Age Groups

#### 3.3.1. Near Convergence Indicator (C′)

**Figure 3:**
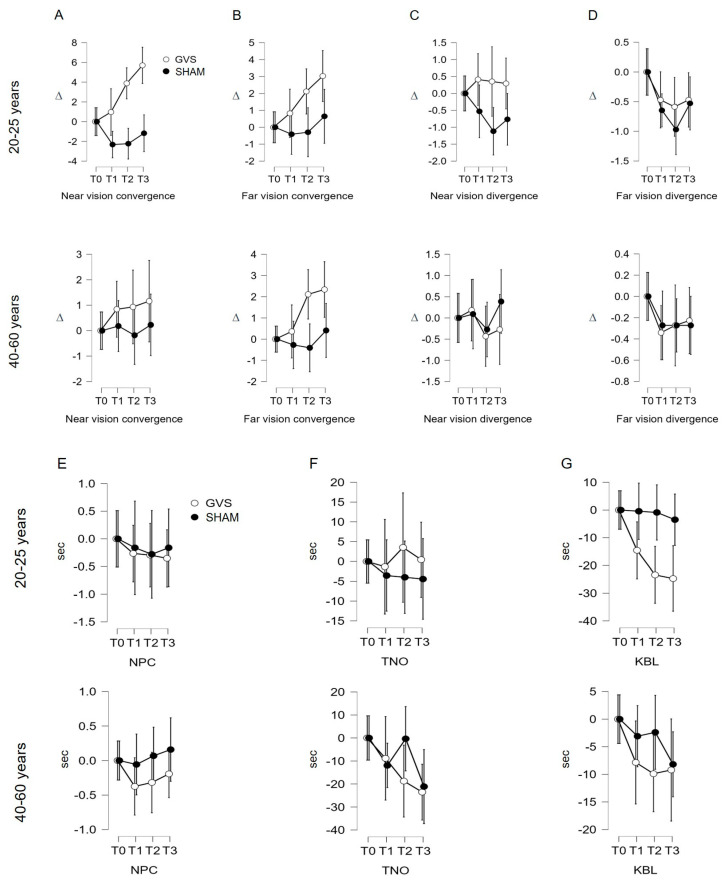
(**A**–**G**). Evolution of measured indicators according to age category. GVS: vestibular galvanic stimulation; sham: stimulation on either side of the C7 spine; NPC: near point of convergence; TNO: stereopsis with graded circle. Distance stereoscopy test (0.40 m); KBL: Kratsa–Barron–Laraudogoitia. Distance stereoscopy test (2.5 m); ∆: diopter, sec: second. The error bars indicate 95% confidence intervals.

Under GVS stimulation, the repeated measures ANOVA shows a significant variation in C′ measurements based on age (F (2.613, 198.569) = 6.327; *p* = 0. 002). The post hoc analysis using Student’s *t*-test shows a significant mean difference for the 20–25 age group between T0 and T2 (*p* = 0.005), T0 and T3 (*p* < 0.001), and T1 and T3 (*p* < 0.001). The interval analysis for the 40–60 age group does not show significant links in the Student’s *t*-test: *p* = 1 among the intervals studied in this group (Figure 3A; Table 2). Under sham stimulation, the repeated measures ANOVA shows a non-significant variation in C′ measurements based on age (F (2.755, 209.389) = 2.251; *p* = 0.089). The post hoc analysis using the Student’s *t*-test does not show a significant mean difference for both age groups (Figure 3A; Table 2).

#### 3.3.2. Analysis of Indicators C, D′, D, NPC, TNO, KBL

Under both GVS and sham stimulation, repeated measures ANOVA does not show significant variations for these seven indicators (Figure 3C–G; Table 2).

## 4. Discussion

In our study, galvanic vestibular stimulation (GVS) improves most of the visuo-oculomotor indicators studied (Table 3).

Our study revealed a beneficial effect of GVS on the indicators C′, C, D, and KBL. The analysis of the control data sets the robustness of the results, ruling out any test-retest effect in all cases except for far divergence (D), which decreases with repeated measures (Figure 2D and Figure 3D; Table 1). The age-stratified analysis concludes that age is a confounding factor only for the C’ indicator, evidencing that the effects of GVS on near convergence occur only in younger subjects (20–25 years). This can be explained by: (1) more efficient neural plasticity and sensory adaptation capacity in younger subjects, allowing more pronounced changes in near convergence and (2) visual system alterations (loss of vergence abilities) and vestibular changes (reduced sensitivity of the system) that limit the effects of GVS in older individuals.

**Figure 4:**
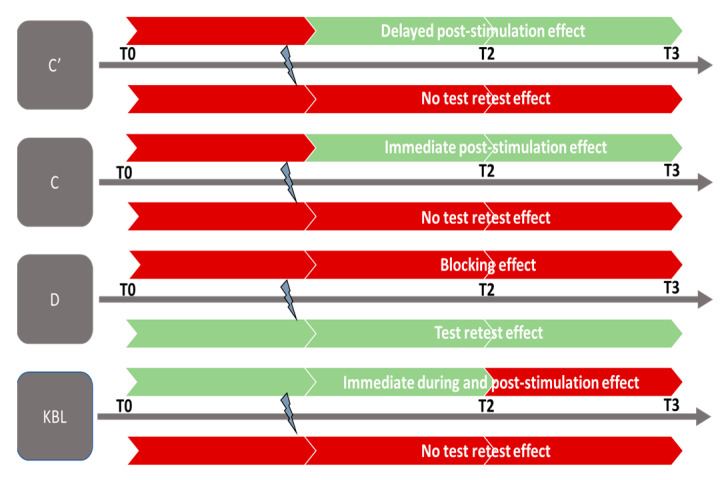
Effect of GVS. C′: near vision convergence; C: far vision convergence; D: far vision divergence; KL: Krats Laraudou test. Colors: 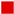 no significant effect; 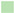 significant effect.

Firstly, the significant increase in far convergence (C) during and after GVS can be interpreted as an improvement in the ability to converge the eyes at a distance in subjects following GVS stimulation. This suggests that the subjects were able to effectively converge their eyes to fixate on distant objects after being subjected to GVS stimulation. It is essential to note that this increase in C (convergence at distance) is observed post-GVS and appears to endure over time, as it persists for up to 15 min after stimulation (Table 3; Figure 4). This suggests that GVS stimulation has both an immediate and lasting effect on the ability to converge at a distance in the study subjects.

Secondly, we observed an increasing trend in near convergence (C′) measurements, demonstrating that GVS influences this indicator during and after its application, seemingly lasting for at least 15 min (Table 3, Figure 4). Similar to far convergence, the results indicate the lasting effect of GVS on this indicator. The increase in C′ values suggests an increase in the amplitude of eye convergence movement during near gaze, indicating that the eyes have a greater capacity to perform this movement when focusing on a nearby object. Nevertheless, this beneficial effect of GVS was only found to be significant for younger subjects.

Furthermore, it is also noteworthy to mention the results of far divergence (D) in the control condition. The shape of the control data curve differs from that of the GVS curve, especially from T1 to T2 (Figure 2D and Figure 3D), and significant values are recorded in the statistical analysis, indicating a significant alteration in this measurement at T2. Assessing the natural variability in an indicator under a control condition allows for a safer interpretation of the results obtained following a particular intervention or stimulation, in this case, GVS. The literature suggests that repeated measurement of vergence can lead to adaptation of the oculomotor system, but it does not directly conclude that far divergence decays with repeated measures [30]. However, in the conditions of this study, the repetition of far divergence measurements deteriorates the D indicator in the control condition. Thus, the dissociation in the curve pattern between the two conditions could imply that GVS may prevent the spontaneous adaptive impact on far divergence during repeated measures.

Finally, we observed a significant decrease in the KBL value during the application of GVS, demonstrating an improvement in far stereopsis during the per-stimulation period (Figure 4). However, it is important to note that the decrease in the KBL value at T2 and T3 (5 and 15 min after GVS stimulation) is visible in the curve in Figure 2G but did not reach statistical significance during the analysis (Table 2 and Table 3. This observation suggests that the effect of GVS on far stereoscopic perception is immediate and may reach a ceiling effect.

Before their cortical integration, visual and vestibular signals are processed together at the level of several subcortical structures, such as the vestibular nuclei (NV) in the brainstem and the thalamus in the diencephalon [31,32]. The vestibulo-ocular reflex (VOR) involves the NV and oculomotor nuclei to maintain stable binocular vision during head and/or body motion. The cerebellum is a key structure that receives vestibular information from the NV to ensure body coordination and balance maintenance, but it also receives visual information (e.g., retinal slips), enabling it to modulate the VOR to stabilize gaze [33,34]. Furthermore, there are subcortical connections that provide tracking or saccade movements during head movements [33,35].

Moreover, the vestibular system interacts with different visual system structures at the other levels: (i) Oculomotor pathways responsible for controlling and coordinating eye movements. The cortico-nuclear tract links cortical associative areas receiving visual information to the vestibular nuclei (NV), allowing coordination between eye movements and body movements to maintain balance [36,37]. (ii) Collicular pathways involving motion receptors and retinal ganglion cells. The superior colliculus is linked to the NV through the tecto-vestibular pathway, enabling precise coordination of eye and body movements in response to visual and vestibular stimuli [38,39]. (iii) Accommodation pathways enabling image clarity regardless of the distance of a fixated object. The link between the oculomotor (II, IV, and V) nucleus and the NV is mainly mediated through the medial longitudinal fasciculus, which maintains precise focus on the object, even during head movements [40]. (iv) Pupillary reflex pathways, which function in coordination to adjust eye focus and pupil size based on environmental visual conditions. The vestibular system detects head rotation movements and sends signals to the nucleus of the trigeminal nerve, which impacts pupil size, triggering constriction of the pupil on the side opposite to the direction of head movement. This is known as the vestibular pupillary reflex, which improves vision sharpness by reducing optical aberrations induced by head movements [40,41].

In our study, the application of low-intensity current in a repeated manner had a primary effect of disrupting the activity of vestibular neurons by modifying the sensory signals transmitted to the NV without causing the appearance of clinical signs. It is important to not confuse the electrophysiological consequences of subthreshold GVS with those of suprathreshold GVS. The latter leads to sufficient neuronal inhibition or excitation to reach the perceptual clinical threshold (vertigo, nausea, and vomiting) and induces measurable behavioral (oculomotor and postural) responses [23,42]. Dlugoiczyk in 2019 and Apba in 2022 [26,27] both proposed an exhaustive review of advances in GVS. Their work addressed cellular and neurophysiological mechanisms as well as clinical applications of this technique. However, how GVS acts on neuronal structures and the most appropriate forms of stimulation for specific applications remain debated. While there are currently few studies in humans that identify the exact electrophysiological modifications after the application of subthreshold GVS, our results show that visuo-oculomotor indicators are sensitive to this stimulation, suggesting an adaptive neuronal process during and after GVS. This neuronal plasticity may allow the system to find a spontaneous resolution to GVS stimulation, explaining the immediate effects observed on visuo-oculomotor indicators. Two studies tested GVS at subliminal and supraliminal intensity levels and recorded induced brain activity using fMRI for each. Bense et al. [43] showed distinct activation of frontal eye fields (FEFs) and the area anterior to FEFs with suprathreshold GVS. Helmchen et al. [44] observed an increase in resting activity of the visual cortex in patients with bilateral vestibular areflexia and a decrease in healthy subjects after subthreshold GVS. The discrepancies in the studies’ conclusions can be attributed to factors such as intensity and form of current used, the type of threshold studied, etc. This allows us to consider a specific spontaneous reorganization of the subliminal signal between vestibular neurons and higher centers of the visuo-oculomotor system. This observation is supported by our results, particularly the persistence of modifications in convergence for both near and far distances even 15 min after subthreshold GVS. Currently, only studies using prolonged stimulation at perceptual thresholds with GVS and IV show reorganization of synaptic circuits up to structural and functional modifications of brain regions involved in processing vestibular and visual information [24,25]. These results offer promising prospects for improving our understanding of the subliminal vestibular error (SVE) signal.

There are some limitations in our study. Although our study design limited measurement bias and confounding factors, our study represents a small sample size from a single center. Further studies are needed with models that focus on other factors such as the duration of GVS exposure, exposure time during the day, and lighting environment to better understand the adaptability of the visuomotor system to subliminal SEV.

## 5. Perspectives and Conclusions

The results of this study highlight the effects of subthreshold GVS on visuo-oculomotor indicators, emphasizing the importance of considering the concept of subliminal vestibular error (SVE) in our understanding of the vestibular system. The existence of an SVE below discrimination thresholds can lead to subtle modifications in visuo-oculomotor coordination mechanisms without manifesting obvious clinical symptoms. This phenomenon finds an interesting parallel with vestibular schwannomas, which can induce a subliminal erroneous signal. In the case of vestibular schwannomas, this configuration is made possible by the slow evolution of the tumor, high plasticity of peripheral vestibular circuitry, and central modulation of detection and discrimination thresholds. Similarly, the subthreshold SVE induced with GVS could engage subtle adaptive neuronal processes, initially localized in the vestibular nuclei and visuo-oculomotor structures, allowing the system to adjust spontaneously to the stimulation. However, further studies will be necessary to confirm our observations and extend them to the population of vestibular patients.

Thus, studying the effects of SVE could be essential for understanding the mechanisms underlying the adaptation and compensation of the vestibular system in response to mild but potentially efficient stimulations on visuo-oculomotor coordination. This improvement in our understanding of SVE could have important clinical implications, particularly for the monitoring and management of patients with subtle complaints related to vestibular dysfunctions. Similar to vestibular schwannomas, where slow progression can initially mask symptoms, SVE could also contribute to compensating for sensory deficits, affecting environmental perception and balance maintenance. The results suggest that the vestibular system possesses robust adaptability to electrical stimulations, even when they do not exceed clinical perception thresholds. These adaptations could manifest as electrophysiological changes, brain reorganization, and adjustments in synaptic connections of visuo-vestibular structures. In the context of our discussion, it will be relevant to explore the implications of the results obtained from GVS on ocular following responses (OFRs) [45,46]. As we analyzed the intricate interactions between visual and vestibular signals, it is noteworthy that the mechanisms underlying neuronal adaptation we observed in response to subliminal GVS stimulation might find parallels with the neuronal responses measured using OFRs. OFRs, being sensitive indicators for visuo-vestibular interactions, could be influenced by similar processes of subtle neuronal plasticity induced with electrical stimulation.

In summary, the study of SVE opens exciting new research perspectives to better understand the complexity of the vestibular system and its interactions with the visual system, paving the way for potential therapeutic and clinical developments aimed at improving the quality of life for patients with vestibular dysfunctions.

## Figures and Tables

**Table 1:** Change of indicators according to the stimulation factor.

Measurements	Stimulation	ANOVA Results	*p*	Significant Post Hoc Analysis
GVS	C′	F (2.613, 198.569) = 10.073	*p* < 0.001	μ(T0)–μ(T2) = −2.407; *p* < 0.002 μ(T0)–μ(T3) = −3.432; *p* < 0.001 μ(T1)–μ(T3) = −2.527; *p* < 0.001
Sham	C′	F (2.755, 209.389) = 2.358	*p* = 0.078	
GVS	C	F (2.772, 210.642) = 13.027	*p* < 0.001	μ(T0)–μ(T2) = −2.116; *p* < 0.001 μ(T0)–μ(T3) = −2.685; *p* < 0.001 μ(T1)–μ(T2) = −1.522; *p* = 0.007 μ(T1)–μ(T3) = −2.092; *p* < 0.001
Sham	C	F (2.492, 189.425) = 1.556	*p* = 0.208	
GVS	D′	F (2.596, 197.322) = 0.460	*p* = 0.683	
Sham	D′	F (2.587, 205.090) = 2.006	*p* = 0.124	
GVS	D	F (2.134, 162.208) = 2.942	*p* = 0.052	
Sham	D	F (2.699, 205.090) = 7.641	*p* = 0.001	μ(T0)–μ(T1) = 0.460; *p* = 0.004 μ(T0)–μ(T2) = 0.622; *p* < 0.001 μ(T0)–μ(T3) = 0.401; *p* = 0.013
GVS	NPC	F (2.236, 169.964) = 2.523	*p* = 0.077	
Sham	NPC	F (1.270, 96.528) = 0.155	*p* = 0.755	
GVS	TNO	F (2.450, 186.182) = 1.281	*p* = 0.282	
Sham	TNO	F (1.797, 136.554) = 2.736	*p* = 0.074	
GVS	KBL	F (2.959, 224.850) = 9.003	*p* < 0.001	μ(T0)–μ(T1) = 11.200; *p* = 0.012 μ(T0)–μ(T2) = 16.634; *p* < 0.001 μ(T0)–μ(T3) = 16.955; *p* < 0.001
C7	KBL	F (2.526, 192.010) = 1.435	*p* = 0.238	

Legend. C: far convergence at 5 m; C′: near convergence at 40 cm; D: far divergence at 5 m; D′: near divergence at 40 cm; NPC: near point of convergence; KBL: Kratsa–Barron–Laraudogoitia; TNO: stereopsis with graded circle, GVS: galvanic vestibular stimulation; C7: C7 spine stimulation. *p* is set to a value < 0.012 after adjustment for post hoc analysis.

**Table 2:** Evolution of monitoring indicators according to age group.

Measurements	Stimulation	ANOVA Results	*p*	Significant Post Hoc Analysis
C′	GVS	F (2.613, 198.569) = 6.327	*p* = 0.002	20–25 years: T0–T2 (*p* = 0.005) T0–T3 (*p* < 0.001) T1–T3 (*p* < 0.001)
C′	Sham	F (2.755, 209.389) = 2.251	*p* = 0.089	
C	GVS	F (2.772, 210.642) = 0.242	*p* = 0.852	
C	Sham	F (2.492, 189.425) = 0.059	*p* = 0.967	
D′	GVS	F (2.596, 197.322) = 0.584	*p* = 0.602	
D′	Sham	F (2.587, 196.629) = 1.360	*p* = 0.258	
D	GVS	F (2.134, 162.208) = 0.338	*p* = 0.720	
D	Sham	F (2.699, 205.090) = 2.296	*p* = 0.086	
NPC	GVS	F (2.236, 1169.964) = 0.351	*p* = 0.728	
NPC	Sham	F (1.270, 96.528) = 0.290	*p* = 0.647	
TNO	GVS	F (2.450, 186.182) = 1.847	*p* = 0.151	
TNO	Sham	F (1.797, 136.554) = 1.709	*p* = 0.188	
KBL	GVS	F (2.959, 224.850) = 1.779	*p* = 0.153	
KBL	Sham	F (2.526, 192.010) = 0.226	*p* = 0.846	

Legend. C: far convergence at 5 m; C′: near convergence at 40 cm; D: far divergence at 5 m; D′: near divergence at 40 cm; NPC: near point of convergence; KBL: Kratsa–Barron–Laraudogoitia; TNO: stereopsis with graded circle, GVS: galvanic vestibular stimulation; sham: C7 spine stimulation. *p* is set to a value < 0.012 after adjustment for post hoc analysis.

**Table 3:** Evolution of indicators according to the stimulation factor across time.

Indicator	Between-Group Variation	Within-Group Variation in the Mean Measurements Taken at Each Time Point (T)												
	T0–T3	*p*	T0–T1	*p*	T0–T2	*p*	T0–T3	*p*	T1–T2	*p*	T1–T3	*p*	T2–T3	*p*
C′	Continuous +	ns	+	s	+	s	+	ns	+	s	+	ns	+	ns
shamC′	no variation	ns	−	ns	−	ns	−	ns	−	ns	+	ns	+	ns
C	Continuous +	ns	+	s	+	s	+	s	+	s	+	ns	+	ns
shamC	no variation	ns	−	ns	−	ns	+	ns	−	ns	+	ns	+	ns
D′	no variation	ns	+	ns	−	ns	−	ns	−	ns	−	ns	+	ns
shamD′	no variation	ns	−	ns	−	ns	−	ns	−	ns	+	ns	+	ns
D	no variation	ns	−	ns	−	ns	−	ns	−	ns	+	ns	+	ns
shamD	Discontinuous	s	−	s	−	s	−	ns	−	ns	+	ns	+	ns
NPC	no variation	ns	−	ns	−	ns	−	ns	+	ns	+	ns	+	ns
shamNPC	no variation	ns	−	ns	−	ns	+	ns	+	ns	+	ns	+	ns
TNO	Continuous −	ns	−	ns	−	ns	−	ns	−	ns	−	ns	−	ns
shamTNO	no variation	ns	−	ns	−	ns	−	ns	+	ns	−	ns	−	ns
KBL	Continuous −	s	−	s	−	s	−	ns	−	ns	−	ns	−	ns
shamKBL	no variation	ns	−	ns	−	ns	−	ns	+	ns	−	ns	−	ns

Legends: *p* < 0.001; ns = *p* > 0.012; +: increasing variation; −: decreasing variation; C: far convergence at 5 m; C′: near convergence at 40 cm; D: far divergence at 5 m; D′: near divergence at 40 cm; NPC: near point of convergence; KBL: Kratsa–Barron–Laraudogoitia; TNO: stereopsis with graded circle. *p* is set to a value < 0.012 after adjustment for post hoc analysis.

## Data Availability

The data presented in this study are available upon request from the corresponding author.

## Appendix A

**Table A1:** Baseline assessment.

Items
Visual acuity measurement (at 5 m: Monoyer chart and at 40 cm: Parinaud chart).
Phoric deviation assessment using the cover test (at 5 m and 40 cm) with horizontal and vertical prism bars.
Evaluation of ocular motility and conjugate eye movements using a fixation target.
Phoric deviation measurement using a Maddox rod (at 5 m and 40 cm).
Measurement of the near point of convergence using a Mawas ruler. Assessment of convergence and divergence fusional amplitudes (at 5 m and 40 cm). Stereo vision examination using the TNO test (at 40 cm) and Laroudoux and Kratz stereograms (at 2.5 m).

**Table A2:** Exclusion criteria.

Items
Heterotropia
Abnormal retinal correspondence (ARC)
Visual acuity less than 10/10 in both eyes
Abnormal fixation (nystagmus)
Abnormal eye movements (paresis, paralysis, alphabetic syndrome) Positive diagnosis of an ocular pathology Positive diagnosis of a general pathology that can impact oculomotor function Positive diagnosis of a neurological or neurodegenerative pathology Positive diagnosis of a vestibular pathology Regular presence of vertigo or motion sickness Ongoing orthodontic and/or orthopedic treatment

**Table A3:** Description of optometric tests used in this study.

Items	Description
Far convergence at 5 meters: C	The subject fixates the light and sees only one, without neutralization. The horizontal prism bar is placed at the base-in position in front of one eye. The operator increases the power of the prism until the subject can no longer fuse. The measurement of convergence is given by the strongest prism that could be compensated, indicated as C + value in diopters (∆). Norms range from 8 to 10 ∆.
Near convergence at 40 cm: C′	Same procedure. The measurement of convergence is given by the strongest prism that could be compensated, indicated as C’ ∆. Norms range from 30 to 40 ∆.
Far divergence at 5 m: D	Same procedure, but the horizontal prism bar is placed base-out in front of one eye: the measurement of divergence is given by the strongest prism that could be compensated, indicated as D ∆. Norms range from 2 to 4 ∆.
Near divergence at 40 cm: D′	Same procedure, but the horizontal prism bar is placed base-out in front of one eye: the measurement of divergence is given by the strongest prism that could be compensated, indicated as D′ ∆. Norms range from 6 to 8 ∆.
Near Point of Convergence: NPC	An object is brought closer until one eye deviates outward, and the NPC (near point of convergence) is measured using a ruler. Its normal value is around 8 to 10 cm from the orbital rim. It is trainable and can be modified voluntarily.
Far Stereoscopic Acuity at 2.5 m: Kratsa-Barron-Laraudogoitia (KBL)	It consists of random red–green dot pattern and is performed using red and green filters. The stereoscopic acuity is measured at 250 s of arc at 5 m and 500 s of arc at 2.50 m. At 5 m, it is a central test, while closer distances involve peripheral fusion. Norms: stereoscopic vision less than 100 s of arc is considered good.
Near Stereoscopic Acuity at 40 cm: Stereopsis with Graded Circle (TNO).	The TNO stereotest consists of six plates (ranging from 480 to 15 s of arc) of anaglyph random-dot stereograms. They should be viewed through red–green glasses. This test measures very fine stereoscopic acuity. Norms: The average stereoscopic acuity in the population is 20 s of arc. For individuals over forty years old, the average value is 58 s of arc.
